# Editorial for the Special Issue on Micromachines for Dielectrophoresis

**DOI:** 10.3390/mi13030417

**Published:** 2022-03-08

**Authors:** Rodrigo Martinez-Duarte

**Affiliations:** Multiscale Manufacturing Laboratory, Department of Mechanical Engineering, Clemson University, Clemson, SC 29634, USA; rodrigm@clemson.edu

Dielectrophoresis (DEP) remains an effective technique for the label-free identification and manipulation of targeted particles ranging from inert particles to biomolecules and cells. Applications are numerous, including clinical diagnostics and therapeutics, advanced manufacturing, electronic displays, and colloidal microrobots. This Special Issue includes 11 novel contributions to the field in multiple aspects from theory to application.

Kale et al. in “Analytical Guidelines for Designing Curvature-Induced Dielectrophoretic Particle Manipulation Systems” [1] present a novel mathematical framework to analyze particle dynamics inside a circular arc microchannel using computational modeling. Their analysis reveals that the design of such devices can be synthesized to three dimensionless parameters and provide validated equations to facilitate the design of curvature-induced DEP systems. The Xuan group at Clemson University also contributes “Passive Dielectrophoretic Focusing of Particles and Cells in Ratchet Microchannels” [2] by Lu et al., which is a fundamental study of the passive focusing of particles in ratchet microchannels using direct current DEP. Via computational modeling and experimentation, they demonstrate how particles were better focused using symmetric ratchet microchannels instead of asymmetric ones and postulate an equation to determine the particle focusing ratio depending on the particle’s DEP and electrokinetic mobilities, channel width, electric field in the constriction, and the number and shape of ratchets.

Hölzel and Pethig contribute “Protein Dielectrophoresis: I. Status of Experiments and an Empirical Theory” [3], where their analysis of the DEP data for 22 different globular proteins revealed that 19 of such works reported protein DEP behavior at an electric field gradient much smaller than the ~4 × 10^21^ V^2^/m^3^ required to overcome the dispersive forces associated with Brownian motion, according to current DEP theory. They note that current DEP theory neglects the contribution of the permanent dipole moment of proteins to the DEP force and present a novel molecular version of the Clausius–Mossotti factor that was derived empirically and, when considered, brings most of the reported protein DEP above the minimum required to overcome dispersive Brownian thermal effects.

Regarding the application of DEP, different groups present their latest results regarding the use of DEP for the concentration of parasites in the context of global health, the use of Janus particles as colloidal microrobots, an integrated microfluidic system for single-cell isolation and retrieval, and the use of complementary metal–oxide–semiconductor (CMOS) fabrication processes to implement a cell viability assay. Keck et al. present “Highly Localized Enrichment of *Trypanosoma brucei* Parasites Using Dielectrophoresis” [4], where titanium electrodes are used to characterize the DEP response of *T. brucei* and enable its rapid enrichment in specific locations on-chip. This work is a step towards facilitating the direct identification of *T. brucei* when attempting to diagnose human African trypanosomiasis, also known as sleeping sickness. Regarding colloidal microrobots, Shen et al. contribute “Frequency Response of Induced-Charge Electrophoretic Metallic Janus Particles” [5], where they describe how electric and magnetic fields can be used to control the direction and speed of Janus particles by exploiting induced-charge electrophoresis (ICEP). Particle motion was characterized through phoretic force spectroscopy across the range 1 kHz–1 MHz, and the authors report a change in direction at ~30 kHz, where particles transition from moving towards their dielectric side below 30 kHz to towards their metallic side above 30 kHz. In “Selective Retrieval of Individual Cells from Microfluidic Arrays Combining Dielectrophoretic Force and Directed Hydrodynamic Flow” [6], Thiriet and co-authors introduce a device for the isolation, retrieval, and off-chip recovery of single cells. Their design uses 3D electrodes embedded in a microfluidic channel to allow for the selective trapping of cells in specific sites through hydrodynamics, and their selective release using a negative DEP force. Cells were then recovered and analyzed off-chip with transcriptional analysis, revealing only a marginal alteration of their molecular profile. In “Dielectrophoretic Immobilization of Yeast Cells Using CMOS Integrated Microfluidics” [7], Ettehad and co-workers validated the use of CMOS-integrated microfluidic devices for the separation and purification of live yeast cells from dead ones using dielectrophoretic forces. This is an important contribution as it further demonstrates the feasibility of using well-established CMOS processes to fabricate DEP devices.

Towards improving the performance of electrowetting (EWD) and electrophoretic (EPD) electronic displays, the industry–academia collaboration between the University of Electronic Science and Technology, South China Academy of Advanced Optoelectronics, and the Shenzhen Guohua Optoelectronics Technology Co. presents “Driving waveform design of electrowetting displays based on an exponential function for a stable grayscale and a short driving time” [8] by Yi et al., and “Driving waveform design of electrophoretic display based on optimized particle activation for a rapid response speed” [9] by He et al. Yi and co-workers postulate an exponential function to drive EWD after studying the impact of the function time constant to reduce flicker and improve the static display performance of EWDs; meanwhile, He and co-workers’ experimental results show that their postulated waveform leads to an improved display quality and a reduction in the flicker intensity in EPDs, when compared to a conventional waveform.

Lastly, important contributions to improving DEP separations are presented. Hawkins et al. in “High sensitivity in Dielectrophoresis separations” [10] critically review multiple ways to improve the sensitivity of DEP-based particle separations. These include combinations of 2D and 3D electrode structures, single or multiple field magnitudes and/or frequencies, and variations in the media suspending the particles. Giesler et al. in “Polarizability-Dependent Sorting of Microparticles Using Continuous-Flow Dielectrophoretic Chromatography with a Frequency Modulation Method” [11] present an improvement in the dielectrophoretic particle chromatography (DPC) of latex particles by exploiting differences in both their DEP mobility and crossover frequencies. To this end, they modulate the frequency of the electric field to induce periodic transitions from positive to negative movement and achieve multiple cycles of particle trap and release.

I would like to thank all the authors for contributing to this first installment of “Micromachines for Dielectrophoresis” as well as all the reviewers whose insightful feedback helped improve the impact of these contributions.
